# An Assessment of Growth Media Enrichment on Lipid Metabolome and the Concurrent Phenotypic Properties of *Candida albicans*

**DOI:** 10.1371/journal.pone.0113664

**Published:** 2014-11-25

**Authors:** Kaushal Kumar Mahto, Ashutosh Singh, Nitesh Kumar Khandelwal, Nitin Bhardwaj, Jaykar Jha, Rajendra Prasad

**Affiliations:** 1 Membrane Biology Laboratory, School of Life Sciences, Jawaharlal Nehru University, New Delhi, India; 2 Department of Biotechnology, Lalit Narayan Mithila University, Kameshwarnagar, Darbhanga, India; University of Geneva, Switzerland

## Abstract

A critical question among the researchers working on fungal lipid biology is whether the use of an enriched growth medium can affect the lipid composition of a cell and, therefore, contribute to the observed phenotypes. One presumption is that enriched medias, such as YPD (yeast extract, peptone and dextrose), are likely to contain lipids, which may homogenize with the yeast lipids and play a role in masking the actual differences in the observed phenotypes or lead to an altered phenotype altogether. To address this issue, we compared the lipids of *Candida albicans*, our fungus of interest, grown in YPD or in a defined media such as YNB (yeast nitrogen base). Mass spectrometry-based lipid analyses showed differences in the levels of phospholipids, including phosphatidylinositol, phosphatidylglycerol, lyso-phospholipids; sphingolipids, such as mannosyldiinositolphosphorylceramide; and sterols, such as ergostatetraenol. Significant differences were observed in 70 lipid species between the cells grown in the two media, but the two growth conditions did not affect the morphological characteristics of *C. albicans*. The lipid profiles of the YNB- and YPD-grown *C. albicans* cells did vary, but these differences did not influence their response to the majority of the tested agents. Rather, the observed differences could be attributed to the slow growth rate of the *Candida* cells in YNB compared to YPD. Notably, the altered lipid changes between the two media did impact the susceptibility to some drugs. This data provided evidence that changes in media can lead to certain lipid alterations, which may affect specific pathways but, in general, do not affect the majority of the phenotypic properties of *C. albicans*. It was determined that either YNB or YPD may be suitable for the growth and lipid analysis of *C. albicans*, depending upon the experimental requirements, but additional precautions are necessary when correlating the phenotypes with the lipids.

## Introduction

Over the last few decades, lipids have been at the center of yeast research. Lipids play a wide range of functions in fungal physiology [1]–[5]. They are known to be key structural components and play a role in processes such as membrane homeostasis, signaling, protein function and sorting, morphogenesis, mitochondrial inheritance, and cell wall integrity [1]–[5]. In yeast, the structural diversity of their lipids is large and thus, equips the cell with rather complex mechanisms for maintaining various cellular functions. Additionally, many lipid groups, such as sterols, sphingolipids (SL) and certain phosphoglycerides (PGL), are structurally very different in yeasts compared to mammalian systems [6]–[9]. And, it is the unique features of these lipids that have allowed the development of many therapeutic approaches against human pathogenic fungi [6]–[9]. Azoles, which inhibit ergosterol biosynthesis by targeting the lanosterol 14α-demethylase (ERG11p), are a classical example, among many other, of antifungal drugs that target yeast lipids [6].

Considering the critical role of lipids in yeast physiology and their clinical implications for antifungal therapies, a large effort has been made to understand their biosynthesis, structure and function [1], [7], [10]. Several genetic, biochemical and biophysical approaches have been used in lipid research [1]. In terms of analyzing the compositional aspect of these lipids, the technology has evolved from classical techniques, such as thin layer chromatography, to high-end techniques, such as mass spectrometry (MS) [1]. The evolution of lipid profiling using high throughput MS-based approaches has proven quite useful in the understanding of yeast lipid metabolism [10].

Our group, along with others, has employed these MS-based methods to understand the lipid metabolism of several human pathogenic fungi [7], [10]–[19]. In our previous studies involving one such fungus, *Candida albicans*, which causes systemic Candidiasis in humans, we explored the role of lipids in its physiology [5,11]–[12], [14]–[18]. For the lipid profiling, we grew *C. albicans* cells either in an enriched medium, such as YPD (yeast extract, peptone and dextrose), or a limited medium, such as YNB (yeast nitrogen base), prior to the lipid extraction [11]–[12,14–18]. Our assumption was that as long as we had a suitable control included, we could compare between the datasets. However, we did observe some marked difference in the profiles on YPD- or YNB-grown *C. albicans* cells in these experiments [11]–[12], [14]–[18]. Recent studies using MS-based lipidomics showed that lipid profiles vary extensively, depending on the physiological state of the yeasts [20]–[25]. In *S. cereviseae*, changes in the lipid profile could be observed following changes in the growth temperature, culture medium, growth phase or carbon source, suggesting that yeast lipids adjust to these conditions by modulating their metabolic state [25]. This observation led to several critical questions regarding *C. albicans* cells grown on YPD or YNB: (i) whether the lipid profile of *C. albicans* is altered when grown in YPD compared to YNB, (ii) if there is a change in the lipids, then what effect do they have on the known phenotypes of *C. albicans*? And (iii) can any of these lipid changes directly affect the susceptibility patterns of frequently used antifungals?

To resolve this issue, we performed a direct comparison of the lipid profiles of *C. albicans* cells grown on YPD or YNB. Our analysis included 9 classes of PGLs, namely phosphatidyl choline (PC), phosphatidyl ethanolamine (PE), phosphatidyl inositol (PI), phosphatidyl serine (PS), phosphatidyl glycerol (PG),phosphatidic acid (PA), lysoPC, lysoPE and lysoPG; 3 classes of SLs namely inositolphosphorylceramide (IPC), mannosylinositolphosphorylceramide (MIPC) and mannosyldiinositolphosphorylceramide (M(IP)_2_C); and sterols. We also evaluated the effect of these lipid changes on the known phenotypes of *C. albicans*. Furthermore, we tested for any contribution that these altered lipid levels may make on the susceptibility patterns of several known antifungals. Together, this study provides us with a complete picture of the differences in the lipid profiles of *C. albicans* cells grown in different media conditions and any direct effect that this change may have on the physiological state of these cells.

## Materials and Methods

### Strains and culture conditions

*C. albicans* strains used in this study is CAI-4. Cells were kept on YPD- (1% yeast extract, 2% glucose, and 2% bactopeptone) or YNB- (0.67% yeast nitrogen base with amino acids, ammonium sulfate, uracil and 2% glucose) plates (HiMedia, Mumbai, India) at 30°C and inoculated in YPD- or YNB- broth. The cells were diluted into 50 ml fresh medium at 0.1 OD at A_600_ (∼10^6^ cells/ml) and grown for 14 h until the cells reached late exponential growth. Cells were washed twice with distilled water prior to lipid extraction.

### Lipid analysis

Lipids were extracted from *C. albicans* cells using a slight modification of the method of Bligh and Dyer as described previously [11], [26]. Briefly, the *C. albicans* cells were harvested at exponential phase from a 50 ml culture and were suspended in 10 ml methanol. 4 g glass beads (Glaperlon 0.40–0.60 mm) were added and the suspension was shaken in a cell disintegrator (B. Braun, Melsungen, Germany) four times for 30 sec with a gap of 30 sec between shakings. Approximately 20 ml chloroform was added to the suspension to give a ratio of 2∶1 of chloroform∶methanol (v/v). The suspension was stirred on a flat-bed stirrer at room temperature for 2 hrs and then filtered through Whatman No. 1 filter paper. The extract was then transferred to a separatory funnel and washed with 0.2 volumes of 0.9% NaCl to remove the non-lipid contaminants. The aqueous layer was aspirated and the solvent of the lipid-containing, lower organic layer was evaporated under N_2_. The lipids were stored at −80°C until analysis.

The mass spectrometry based lipidome analysis employed in the present paper draws from, and is consistent with our earlier work [11], [12], [15]. For lipid profiling, the following quantities of internal standards were added to the lipid extracts: 0.6 nmol di12:0-PC, 0.6 nmol di24:1-PC, 0.6 nmol 13:0-LysoPC, 0.6 nmol 19:0-LysoPC, 0.3 nmol di12:0-PE, 0.3 nmol di23:0-PE, 0.3 nmol 14:0-LysoPE, 0.3 nmol 18:0-LysoPE, 0.3 nmol di14:0-PG, 0.3 nmol di20:0(phytanoyl)-PG, 0.3 nmol 14:0-LysoPG, 0.3 nmol 18:0-LysoPG, 0.3 nmol di14:0-PA, 0.3 nmol di20:0(phytanoyl)-PA, 0.2 nmol di14:0-PS, 0.2 nmol di20:0(phytanoyl)-PS, 0.23 nmol 16:0–18:0-PI, 0.16 nmol di18:0-PI, 4.6 nmol di15:0-DAG (Avanti Polar Lipids, Alabaster, AL). Then these samples were suspended in chloroform/methanol/300 mM ammonium acetate in water (300/665/35,v/v/v) in a final volume of 1.4 ml. These lipid extracts were directly introduced by continuous infusion into the ESI source on a triple quadrupole MS (API 4000, Applied Biosystems, Foster City, CA).

PGL species were detected with the following scans: PC and LysoPC, [M+H]^+^ ions in positive ion mode with Pre 184.1; PE and LysoPE, [M+H]^+^ ions in positive ion mode with NL 141.0; PA, [M+NH_4_]^+^ in positive ion mode with NL 115.0; PG, [M+NH_4_]^+^ in positive ion mode with NL 189.0 for PG; PI, [M+NH_4_]^+^ in positive ion mode with NL 277.0; PS, [M+H]^+^ in positive ion mode with NL 185.0; LysoPG, [M – H]^−^ in negative mode with Pre 152.9. The collision gas pressure was set at 2 (arbitrary units (au)). The collision energies, with nitrogen in the collision cell, were +40 V for PC, +28 V for PE, +25 V for PA, +22 V for PG, PI and PS, and −57 V for LysoPG. Declustering potentials were +100 V for PC, PE, PA, PG, PI, and PS, and −100 V for LysoPG. Entrance potentials were +14 V for PC, PA, PG, PI, and PS, +15 V for PE, and −10 V for LysoPG. Exit potentials were +14 V for PC, PA, PG, PI, and PS, +11 V for PE, and −14 V for LysoPG.

SL species were detected with the following scans: IPC, [M−H]^−^ ions in negative ion mode with Pre 259; MIPC, [M−H]^−^ ions in negative ion mode with Pre 421; M(IP)2C, [M−H]^−^ in negative mode with Pre 663.1. The internal standard, 16:0–18:1-PI, was detected with [M−H]^−^ in negative ion mode with Pre 241.0. The collision energies, with nitrogen in the collision cell, were −72 V for IPC, −80 V for MIPC, and −75 V for M(IP)_2_C. The declustering potential was −180 V for IPC, MIPC, and M(IP)_2_C. The exit potential was −15 V for IPC, MIPC, and M(IP)_2_C.

SE species were detected with the following scans: 16:1, 16:0, 18:3, 18:2, 18:1 and 18:0 -containing species, were detected as [M+NH_4_]^+^ in positive ion mode with NL 271.2, 273.2, 295.2, 297.2, 299.2 and 301.2 respectively. The internal standard, di15:0-DAG was detected with [M+NH_4_]^+^ in positive ion mode with NL 259.2. The collision energy, with nitrogen in the collision cell, was +25 V. The declustering potential was +100 V. The exit potential was +12 V.

The source temperature (heated nebulizer) was 100°C, the interface heater was “on”, and +5.5 kV or −4.5 kV were applied to the electrospray capillary during these analyses. PGLs, SLs and SEs were quantified exactly as described previously [11], [12], [15].

### Growth assessment during the drug treatment

The response of the *C. albicans* cells to various drugs in either YPD or YNB was monitored by adopting the minimum inhibitory concentration (MIC) method. 0.001 OD cells were inoculated in each well and growth was monitored with or without drugs after 48 h. The following are the different classes of antifungal agents used in this study: i) phospholipid analogue (Miltefosine, MLT); ii) sphingolipid biosynthesis inhibitor (Myriocin, MYR); azole (Fluconazole, FLC; Miconazole, MICO; Ketoconazole, KETO; Itraconazole, ITRA); iii) polyenes (Amphotericin B, AMPB; Nystatin, NYS); iv) protein biosynthesis inhibitor (Cycloheximide, CYCLO); cell wall perturbing agents (Calcofluor white, CW; Congo red, CR; TritonX100, TX-100; Sodium dodecylsulfate, SDS). The chemicals used in these experiments were purchased from Sigma Chemical Co. (St. Louis, MO, USA). The MICs against the *C. albicans* strain were determined by a broth microdilution using two-fold serial dilutions in YPD or YNB medium, as described in method M27-A3 by the Clinical and Laboratory Standards Institute (CLSI, formerly NCCLS, USA) [27].

### Statistical Analysis

All of the experiments were performed in two or more replicates and are represented as the mean ± standard error of the mean (SEM). Statistically significant lipid changes were highlighted by pattern recognition tools, such as principal component (PCA) and discriminant analysis, using the software SYSTAT, version 10 (Systat Software Inc., Richmond, CA, USA). The processing of the datasets for the PCA was done as previously described [11]. Any statistically significant differences were identified using Student's *t*-test. A significance level of 0.05 was employed.

## Results

### Lipid profiling of the YPD- or YNB-grown *C. albicans* cells

For the lipid profiling, three major lipid groups were analyzed: PGLs, SLs and sterols [12]. These lipid groups have been shown to play a crucial role in the physiology of *C. albicans*, affecting a broad range of cellular processes, such as growth, morphogenesis, acquired resistance to drugs, virulence and pathogenesis [5]. The lipids extracted from the YPD- or YNB-grown *C. albicans* were analyzed using the ESI-MS/MS methods established previously [11], [12], [15].

#### Effect on the PGL composition

**Figure 1:**
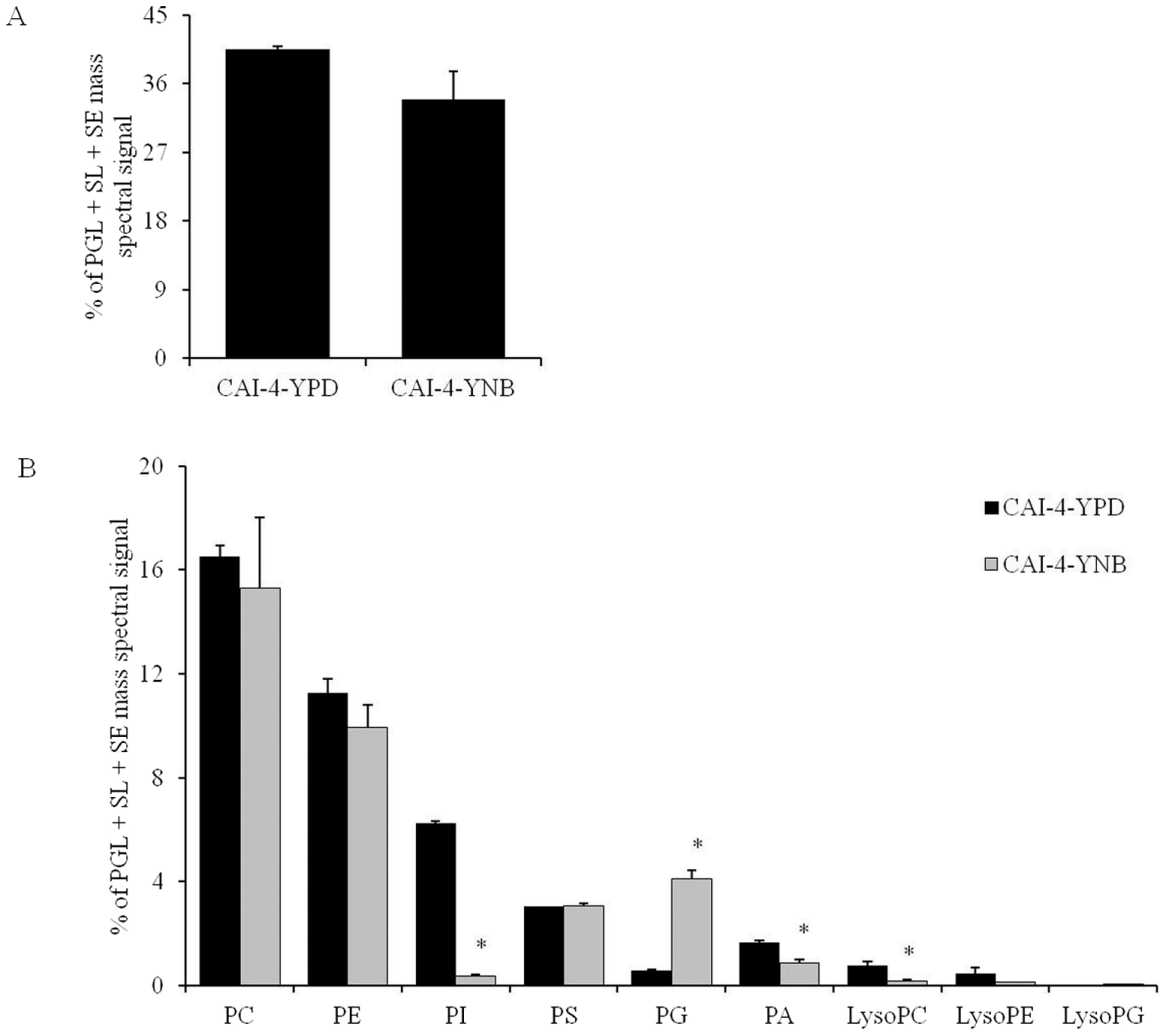
PGL composition of *C. albicans* cells grown in different medium. A) Total PGLs (as normalized total PGL+SL+SE mass spectral signal). B) Relative abundance of PGL classes (as % of normalized total PGL+SL+SE mass spectral signal). Values are mean of 2 independent analyses (n = 2). Data can be found in Supplementary Sheet S1. “*” indicates that *p*-value is <0.05.

Nine major PGLs of *C. albicans* were analyzed [12], namely PC, PE, PI, PS, PG, PA, lysoPC, lysoPE and lysoPG. There was no significant difference in the overall PGL content (Fig. 1A); however, an increased abundance in the PG (7.1-fold) content was observed in the YNB-grown cells compared to the YPD-grown cells (Fig. 1B). Furthermore, a decrease in PI (17.5-fold), PA (1.9-fold), and lysoPC (4.6-fold) was observed in the YNB-grown cells compared to the YPD-grown cells (Fig. 1B).

#### Effect on the SL composition

**Figure 2:**
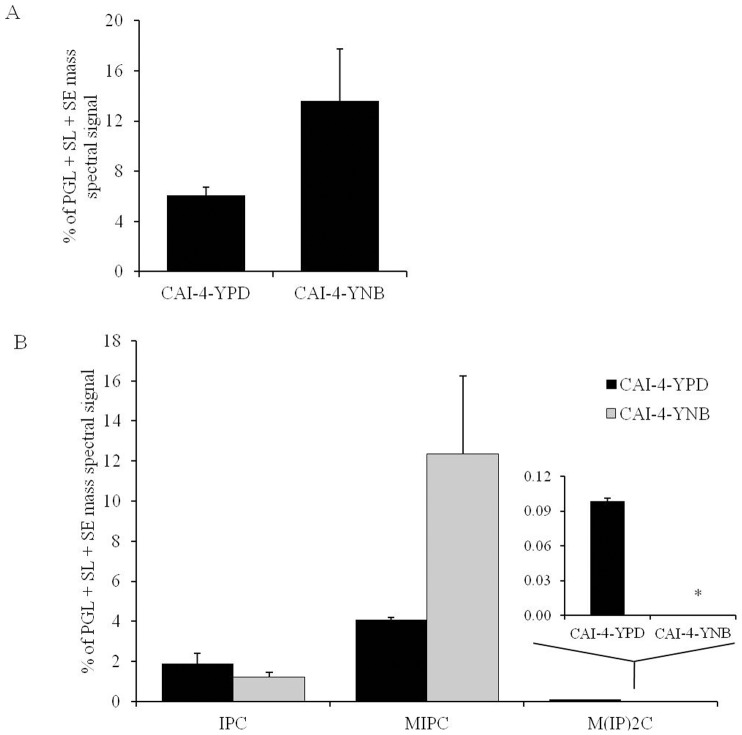
SL composition of *C. albicans* cells grown in YPD or YNB. A) Total SLs (as normalized total PGL+SL+SE mass spectral signal). B) Relative abundance of SL classes (as % of normalized total PGL+SL+SE mass spectral signal). Values are mean of 2 independent analyses (n = 2). Data can be found in Supplementary Sheet S1. “*” indicates that *p*-value is <0.05.

Three major SLs of *C. albicans* were analyzed [12], namely IPC, MIPC, and M(IP)_2_C. Although the overall SL content showed a trend towards depletion in the YNB-grown cells, this change was not statistically significant (Fig. 2A). Similarly, the changes in IPC and MIPC were not statistically significant (Fig. 2B). M(IP)_2_C was not detected in the YNB-grown cells but was present in the YPD-grown cells (Fig. 2B).

#### Effect on the sterol composition

**Figure 3:**
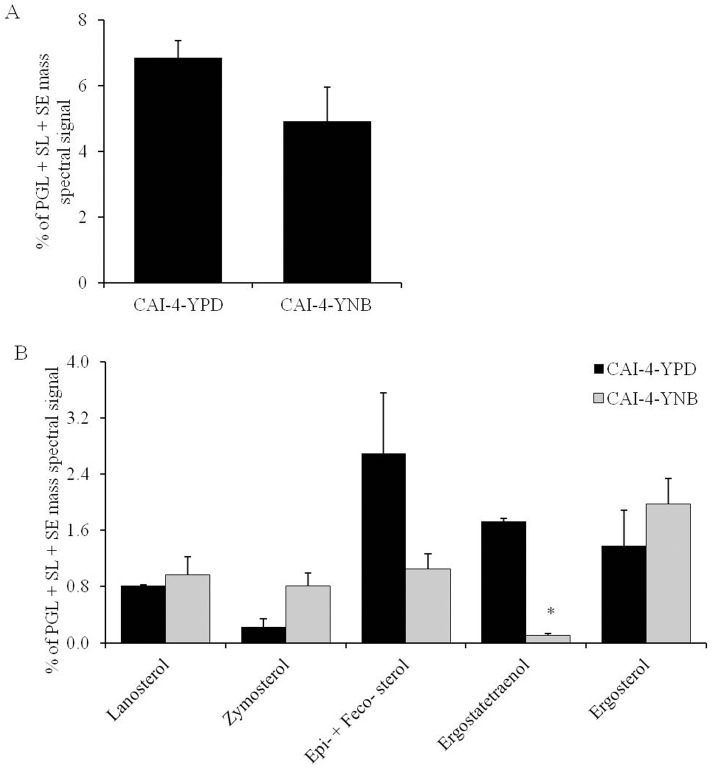
Sterol composition of *C. albicans* cells grown in YPD or YNB. A) Total SEs (as normalized total PGL+SL+SE mass spectral signal). B) Relative abundance of SE classes (as % of normalized total PGL+SL+SE mass spectral signal). Values are mean of 2 independent analyses (n = 2). Data can be found in Supplementary Sheet S1. “*” indicates that *p*-value is <0.05.

Six major sterol intermediates of *C. albicans* were analyzed [12], namely lanosterol, zymosterol, episterol, fecosterol, ergostatetraenol and ergosterol. The overall sterol content remained unchanged between the YPD- and YNB-grown cells (Fig. 3A). A decrease in the ergostatetraenol content (15.8-fold) was observed in the YNB-grown cells compared to the YPD-grown cells (Fig. 3B). No significant alteration in any of the other sterol intermediates was observed (Fig. 3B).

#### Alterations in the lipid species

**Figure 4:**
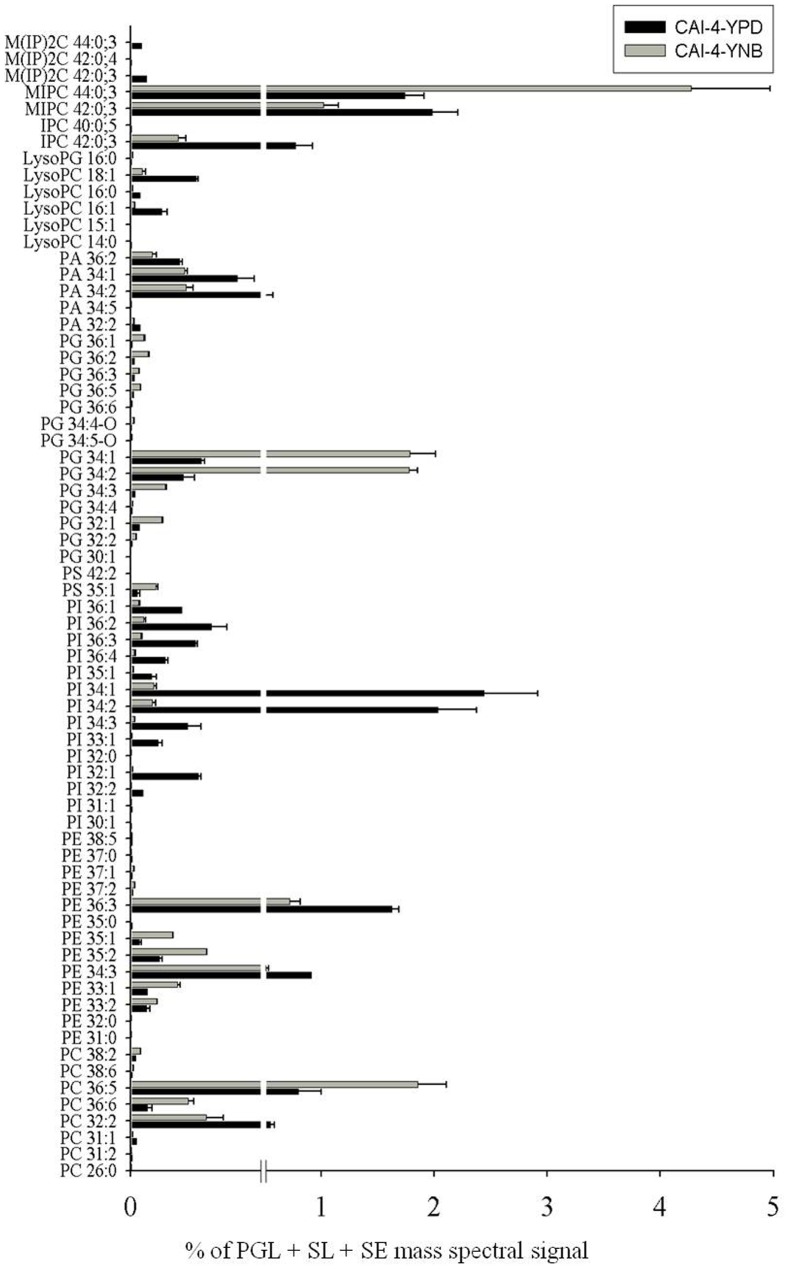
Molecular lipid species composition of *C. albicans* cells grown in YPD or YNB. Data is represented as % of total PGL+SL+SE mass spectral signal normalized to the internal standards. Values are means ± SEM (n = 2). Data can be found in Supplementary Sheet S1. Only significant changes where *p*-value is <0.05 are depicted in this figure.

We analyzed the changes at the lipid-species level for various classes of lipids. Among over 240 lipid species that were targeted in our analyses (Supplementary Sheet S1), 70 species showed significant differences (Fig. 4). Specifically, 35 lipid species showed a lower abundance and 35 lipid species showed a higher abundance in the YNB-grown cells when compared to the YPD-grown cells. The majority of the low-abundance species in the YNB-grown cells belonged to PI, PA, lysoPC and SLs, while those with a higher abundance belonged to the PI, PG and lysoPG classes (Fig. 4). Both PC and PE showed mixed differences.

**Figure 5:**
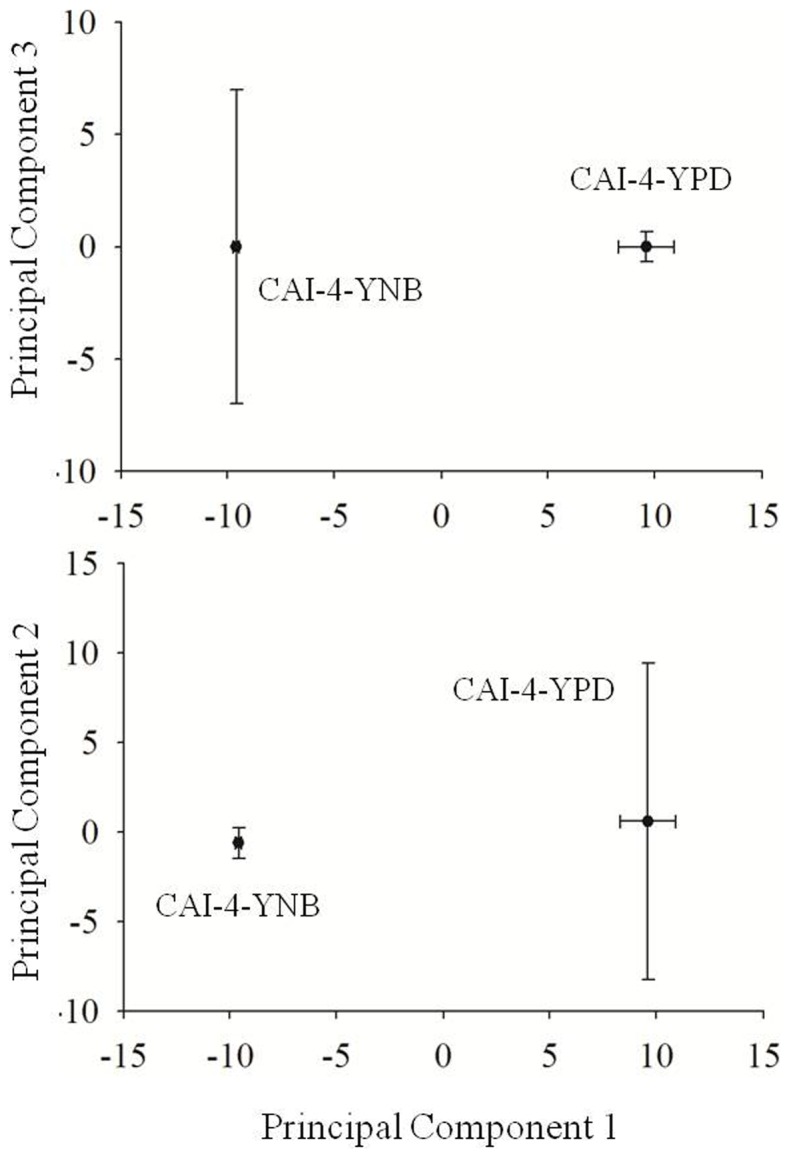
PCA analysis of lipid species of *C. albicans* cells grown in YPD or YNB. The figure shows the 2D- PCA score plots, where the scores for the first three principal components, explaining >40% of the variance, are plotted. A) Principal component 1 versus Principal component 2. B) Principal component 1 versus Principal component 3. Each point in the PCA plot represents the principal component score of the individual replicate. Data for the PCA analyses was taken from Supplementary Sheet S1. Loading values are indicated in Table 1.

The statistical significance of these lipid species changes was tested using a principal component analysis (PCA) [28]. The highest and lowest loading scores, which determine each of the principal components, are listed in Table 1. In the plot of principal component 1 versus principal component 2 (Fig. 5), principal component 1 describes the distinction between the lipids of the YPD- and YNB-grown cells. The principal component 1 loadings (Table 1), suggest that the PG and PE species are crucial for the segregation of these groups (viz. YPD or YNB) along the negative principal component 1 axis, while the content of the M(IP)_2_C, lysoPC and PI species are crucial for the segregation of these groups along the positive principal component 1 axis. Principal component 2 also distinguishes between the YPD- and YNB-grown cells (Fig. 5). The lowest loading scores for principal component 2 depict the low abundance of the PE, PS and IPC species in the YNB-grown cells, while the highest loading scores represent increasing trends in the amounts of several PE, PS and lyso-lipid species. Notably, the lipid species depicted in the loadings of principal component 1 represent the most statistically significant changes between the lipid species in the YPD- and YNB-grown cells.

**Table 1:** Loadings of principal components 1 and 2 from PCA analysis of lipids species of *C. albicans* cells grown in different medium.

Lipid	Principal	Lipid	Principal
Species 1	Component 1	Species 2	Component 2
*12 Lowest loading values*			
PE 35:2	−1.000	PE 32:1	−0.969
PE 35:1	−1.000	IPC 42:0;4	−0.933
PG 36:5	−0.999	PS 34:1	−0.931
PG 34:4-O	−0.998	PS 30:0	−0.854
PG 34:3	−0.996	PE 30:2	−0.815
PG 34:2	−0.995	PS 38:4	−0.805
PG 34:5-O	−0.995	PE 38:3	−0.787
PG 36:2	−0.994	PE 34:1	−0.779
PG 32:1	−0.990	PE 38:2	−0.771
PE 33:1	−0.989	Ergosterol ester	−0.768
PA 34:5	−0.987	IPC 40:0;4	−0.754
PS 42:2	−0.984	PS 30:2	−0.754
*12 Highest loading values*			
PA 32:2	0.989	PE 42:2	0.897
M(IP)_2_C 42:0;3	0.990	LysoPE 16:0	0.899
LysoPC 15:1	0.990	PS 36:4	0.907
PE 36:3	0.991	LysoPC 17:0	0.912
PC 31:1	0.991	PS 34:4	0.915
Ergostatetraenol ester	0.992	PS 32:2	0.955
PI 36:1	0.992	PE 36:6	0.959
LysoPC 14:0	0.992	PS 34:2	0.967
M(IP)_2_C 44:0;3	0.994	PS 36:3	0.980
PE 34:3	0.996	LysoPE 17:1	0.986
PI 32:2	0.997	PS 36:5	0.990
PI 32:1	1.000	PE 31:1	0.990

The 12 highest and 12 lowest values are indicated.

### Effects on growth and morphology

**Figure 6:**
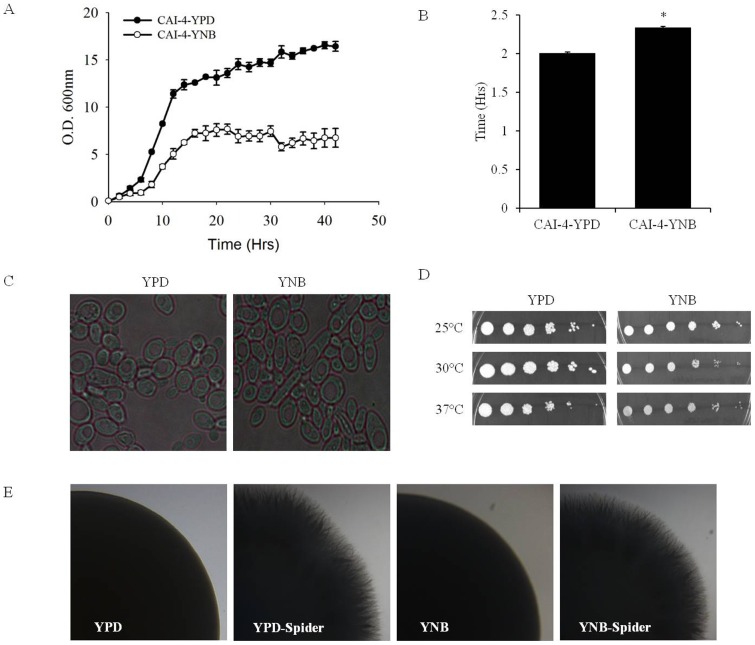
Growth phenotypes of *C. albicans* cells grown in YPD or YNB. A) Growth curve of CAI-4 cells grown in YPD and YNB medium. B) Generation time of CAI-4 cells grown in YPD or YNB medium. C) Cell morphology of CAI-4 cells grown in YPD or YNB medium. D) Spot assays showing growth of CAI-4 cells grown in YPD or YNB medium at variable temperatures. E) Hyphae formation of CAI-4 cells grown in YPD or YNB. *Candida* strains were cultured in YPD or YNB medium at 30°C as described in previously [11], [18]. To test the hyphae formation YPD or YNB grown cells were re-grown on spider agar plates at 37°C for 5 days. Values are means ± SEM (n = 3). “*” indicates that p-value is <0.05.

Several studies have documented the growth and morphological phenotypes of *C. albicans* cells grown in YPD or YNB [11]–[12], [14]–[16], [18], [29]–[33]. We performed a re-evaluation of these phenotypes under our conditions as a necessary precaution. The growth of *C. albicans* was slower in YNB compared to the YPD-grown cells (Fig. 6A). This also corroborated with a higher doubling time (2.34 hrs) in YNB compared to the YPD-grown cells (2.01 hrs) (Fig. 6B). However, there were no visible structural or morphological abnormalities in the yeast cells grown in either the YPD or YNB (Fig. 6C). The cells grew normally when the temperature was changed (Fig. 6D); yet, as expected, the growth in YNB was slower than in YPD at all of the tested temperatures (Fig. 6D). Additionally, all of the cells grown in either YPD or YNB formed adequate hyphae (Fig. 6E).

### Response to known antifungal agents

The mechanism of action of many *in vitro* tested and clinically used antifungal drugs directly or indirectly involves lipids or their biosynthetic pathways [34]–[37]. These include lipid inhibitors, cell wall perturbing agents and protein biosynthesis inhibitors [34]–[40]. We tested whether the changes in the lipid profile due to growth on either YPD or YNB would have an effect on the susceptibility patterns of these drugs.

#### Lipid inhibitor

**Figure 7:**
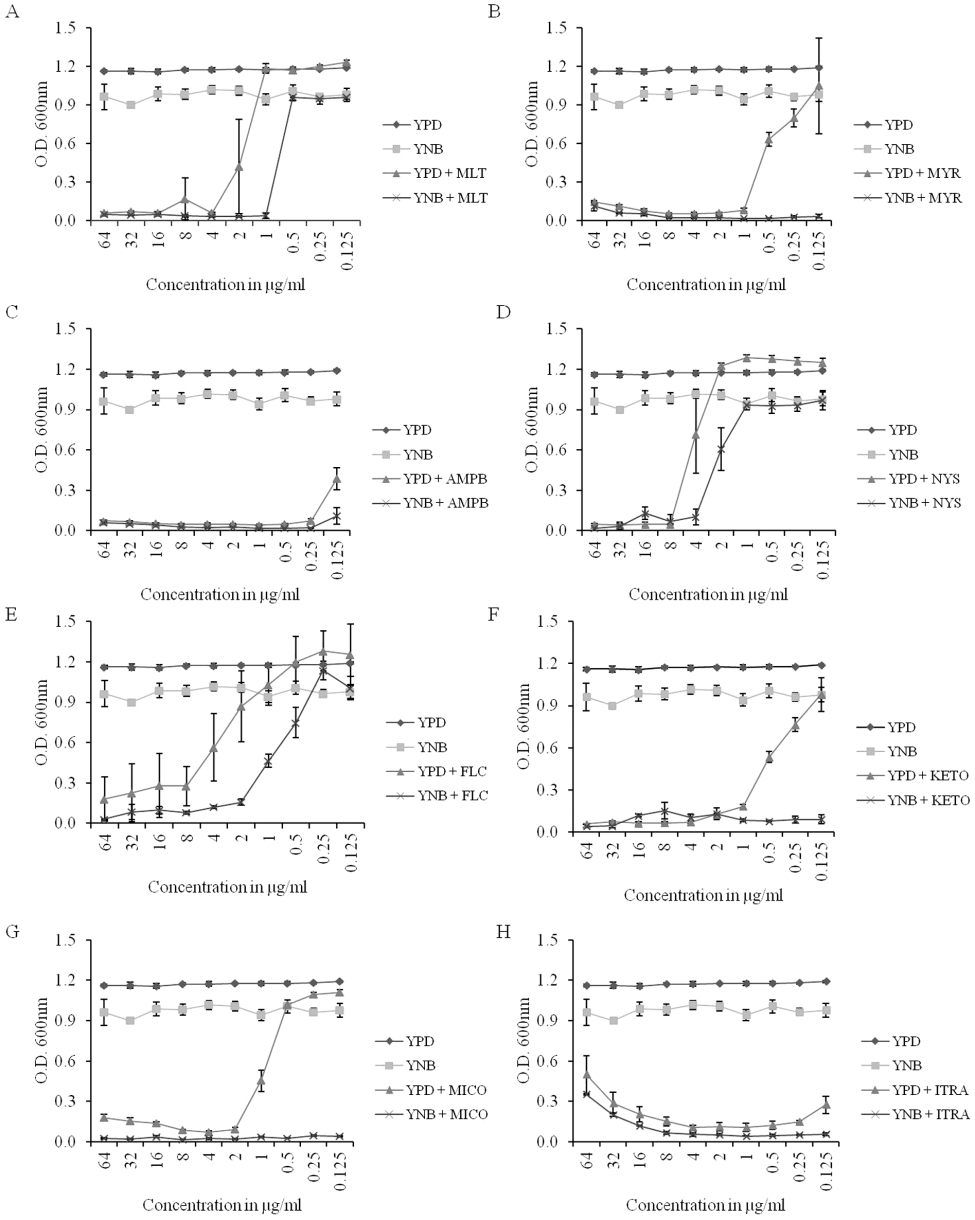
Effect of lipid biosynthesis inhibitors on *C. albicans* cells grown in YPD or YNB. A) Effect of PC analogue: MLT. B) Effect of phytoceramide biosynthesis inhibitor: MYR. C–D) Effect of ergosterol binding drugs: AMPB and NYS. E–F) Effect of ergosterol biosynthesis inhibitors: FLC, MICO, KETO and ITRA. Values are means ± SEM (n = 4). YPD and YNB control datasets are same for the subfigures A–H.

MLT, a known PC analog, affects the overall PGL homeostasis. MLT showed a higher inhibition rate in YNB compared to the YPD-grown cells (Fig. 7A); however, this change may be due to the slow growth of the cells in YNB compared to YPD (Fig. 7A). Similarly, the SL biosynthesis inhibitor, MYR, also showed a higher inhibition rate in YNB compared to the YPD-grown cells, which can also be attributed to the slow growth of the cells in YNB compared to YPD (Fig. 7B). The ergosterol sequestering drugs, AMPB and NYS, showed faster growth inhibition in YNB compared to the YPD-grown cells, which again may be attributed to the slow growth rate of the YNB-grown cells (Fig. 7C and 7D). Similar results were observed for the azoles, which target ERG11 of the ergosterol biosynthetic pathway. Both FLC and KETO showed a higher growth inhibition in YNB, which is likely attributed to the slow growth of the cells in this medium (Fig. 7E and 7F). Although the cells showed a high susceptibility for both MICO and ITRA, the pattern remained the same as that observed for FLC and KETO (Fig. 7G and 7H).

#### Cell wall perturbing agents

**Figure 8:**
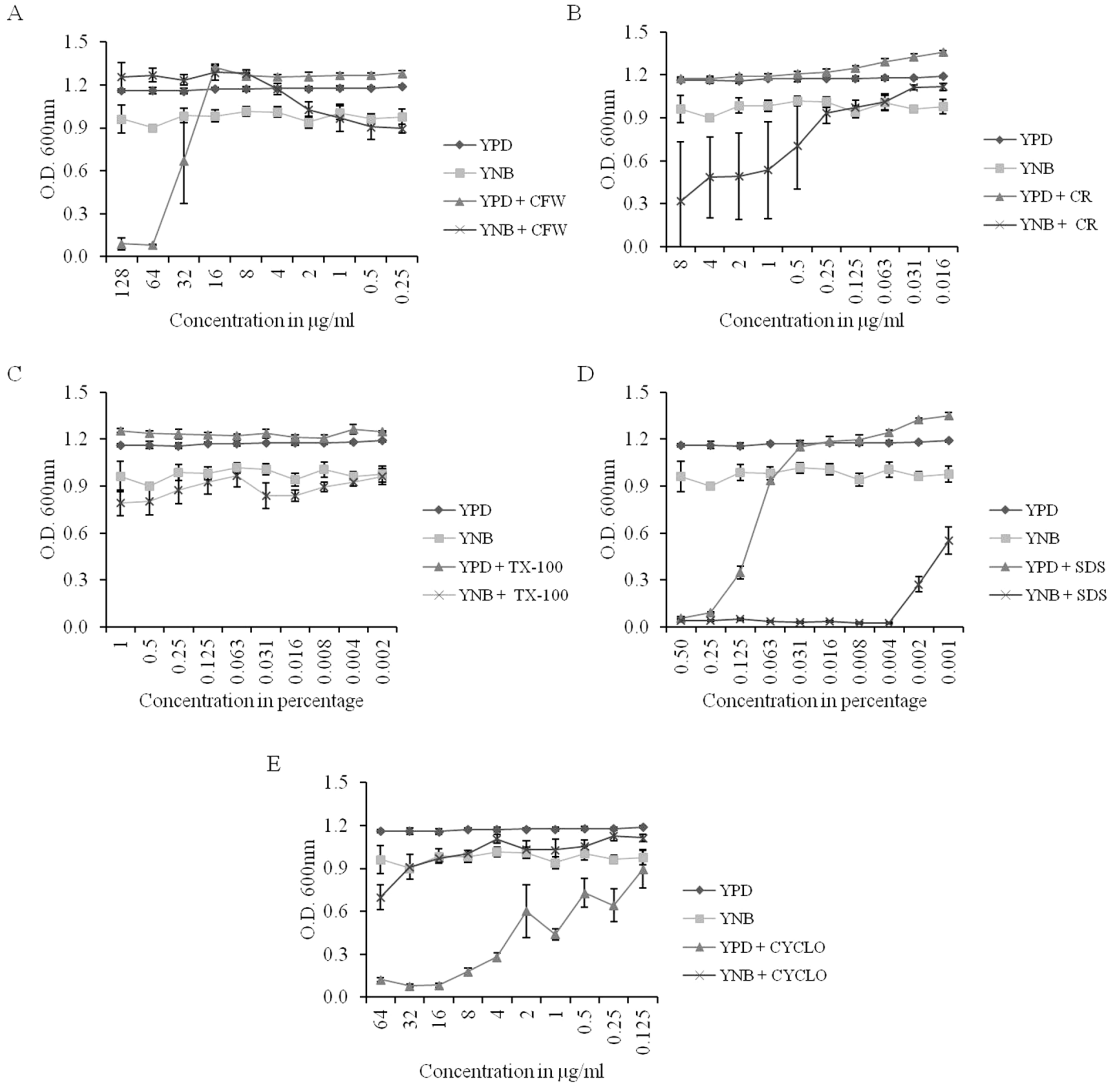
Effect of cell wall perturbing agents and protein biosynthesis inhibitors on *C. albicans* cells grown in YPD or YNB. A) CW. B) CR. C) TX-100. D) SDS. E) CYCLO. Values are means ± SEM (n = 4). YPD and YNB control datasets are same for the subfigures A–E.

CW, which targets the chitin content of the fungal cell wall, showed a higher growth inhibition in YPD but a low activity in the YNB-grown cells (Fig. 8A). CR, which targets the β-1,3-glucans in the fungal cell wall, showed a slow growth inhibition in YNB-grown cells (Fig. 8B); yet, the observed difference between YPD and YNB may be due to the difference in the growth rates. TX-100, a non-ionic surfactant, showed no growth inhibition in either YPD or YNB. Instead, the YPD-grown cells appeared to be healthier following the treatment (Fig. 8C). On the converse, SDS, an anionic surfactant, showed considerable growth inhibition in both the YPD- and YNB-grown cells (Fig. 8D), but like most of the other compounds tested, the higher SDS susceptibility in YNB could be due to the slower growth rate.

#### Protein biosynthesis inhibitors

We observed that CYCLO showed inhibition in the YPD-grown cells but had minimal effect on the YNB-grown cells (Fig. 8E).

## Discussion

In general, the lipids in fungi play a crucial role in their physiology [1]–[5]. Among pathogenic fungi, several lipid classes remain crucial for the maintenance of pathogenesis and virulence [18], [41]–[44]. Therefore, these lipid classes are the prime targets to develop antifungal therapies. The development of high-throughput techniques, such as MS, has equipped us with a quick, sensitive and robust methodology to analyze these lipids [1], [7], [10]–[11]. We are able to not only quantify various lipid classes but also the molecular species of the lipids therein. In non-pathogenic yeasts like *S. cereviseae*, studies have shown the occurrence of remarkable changes within the lipidome by changing the culture conditions [25]. Therefore, it is important to assess the effect of these lipid metabolic changes on the phenotypic properties of the fungi used in a study.

*C. albicans* is one of the most predominant human pathogenic fungi [45]. In the past, several studies have demonstrated the importance of lipids to this fungus. YPD and YNB are the two most frequently used mediums for growing this fungus *in vitro* [11]–[12], [14]–[18]. In the present study we have evaluated the lipid profile differences of *C. albicans* induced by growth in YPD, an enriched medium, or YNB, a limited medium. We analyzed 13 lipid classes, including the lipid species therein, and found that there is a large-scale remodeling of the lipids between YPD- versus YNB-grown *C. albicans* cells. Significant differences were found in the PI, PG, PA, lysoPC, MIPC, M(IP)_2_C and ergostatetraenol lipids. Many high-abundance lipid classes, such as PC, PE, PS, IPC and ergosterols, were resistant to any change and their levels did not alter significantly between the YPD- or YNB-grown cells. However, the lipid species did show massive profile changes. With almost 70 lipid species showing differences, it is clear that the lipid profile of the YPD-grown cells is significantly different from the YNB-grown cells.

To what extent these lipid perturbations might affect or contribute to the physiological behavioral/phenotypic properties in *C. albicans* still requires further assessment. Our data showed that YNB-grown *C. albicans* cells have a longer doubling time than the YPD-grown cells. Additionally, the YNB-grown cells showed a slower growth rate under all of the tested temperatures. These data are consistent with earlier reports demonstrating the doubling time of *C. albicans* in different growth media and its temperature-dependent growth [30]. Differences in the growth phases and culture conditions are known to affect the lipid metabolic state of yeast [20]–[25]. Therefore, our observation of altered lipid profiles upon changing the growth medium from YPD to YNB is expected and remains consistent with earlier reports. The fact that we see no abnormality in the morphological characteristics, such as cell shape or structure, or hyphae formation patterns, suggests that *C. albicans* cells are able to maintain at least these phenotypes despite the lipid changes that are observed due the change of media. Also, a recent study has shown that alteration in lipid composition can affect the mating processes in yeasts grown [46]. Mating phenotypes have been studied in YNB- and YPD- grown cells. Visually, both shmoo formation and polarization of the cells appear to be similar in YPD and YNB [46]–[47]. However, differences may exist in the rate at which these processes occur. Another study showed that YNB- grown cells show lower budding compared to YPD- grown cells and this might explain the slow doubling time observed in YNB grown *C.* albicans [48]. Although these differences in budding/mating phenotypes may exist, but at this point it is difficult to determine whether or up to what extent these changes are a result of altered lipid biosynthesis upon growing *C. albicans* in YPD or YNB.

In last 10 years, numerous studies have shown the importance of lipids to the physiology of *C. albicans*, including raft mediated signaling, protein trafficking, vacuoles, the mitochondria and the cell wall [5], [35], [44]. The presence of fungal-specific lipids, such as ergosterol and mannosylated SLs, presents us with a therapeutic target to kill fungi such as *C. albicans* [35], [41], [43]. A number of laboratory and/or clinical antifungals are known to be directly or indirectly linked to the lipid metabolism of fungi. Using several available antifungals, we tested if the alterations in the lipid profiles of the YPD- versus YNB-grown cells would somehow alter the growth phenotype of *C. albicans* upon drug treatment in the two media. The data showed that *C. albicans* cells showed growth inhibition in both YPD and YNB against the majority of the lipid inhibitors, including MLT, MYR, AMPB, NYS, FLC, KETO, MICO and ITRA. The growth inhibition in the YNB-grown cells appears to be higher than in the YPD-grown cells. However, this change can be accounted for by the low growth rate of *C. albicans* in YNB compared to YPD. This data is also supported by the fact that the PC, SL and ergosterol content, which are the target molecules for these drugs, did not change significantly between the YPD- or YNB-grown *C. albicans* cells (Fig. 1A, 2 and 3B). Therefore, we conclude that although the lipids are affected by changes in the growth media, these changes do not directly contribute to the observed effect of these inhibitors. Apparently, the large scale changes observed at the level-lipid species (Fig. 4) also appear to be compensatory to the growth medium used and may or may not affect the observed phenotypes of *C. albicans*.

Recent studies have demonstrated a clear link between lipids and the cell wall in *C. albicans* [15], [42]. We observed that CW, a cell wall perturbing agent, caused growth inhibition in the YPD-grown cells but not in the YNB-grown cells. A recent study demonstrated that PG, an important mitochondrial lipid, is essential to cell wall integrity in *C. albicans* [15]. *C. albicans* strains with a low PG content have weaker cell walls and show higher susceptibility towards cell wall-perturbing agents [15]. Therefore, the fact that we observed growth inhibition upon CW treatment in the YNB-grown cells could be explained by the higher PG content of these cells. This data presents an example of a media-induced lipid change that is compensated for by a growth phenotype in *C. albicans*. Other cell wall perturbing agents, such as CR, TX-100 and SDS, showed growth inhibition patterns that appear to be unlinked with the lipid metabolic state of the YPD- or YNB-grown cells.

Protein biosynthesis inhibitors present a class of antifungals that do not directly affect lipid metabolism [39], [40]. We observed that CYCLO showed growth inhibition only in YPD and not in the YNB-grown *C. albicans* cells. We are unable to comment whether the changes in the lipid composition could be linked to this observed change, but there was a noticeably different response between the two media.

Lipids remain one of the most extensively studied molecules in fungi. In *C. albicans* alone, a tremendous amount of literature is available showing the importance of lipids in this fungus. In this study, we clearly showed a remarkable change occurs in the lipids of *C. albicans* when grown in either YPD or YNB. We also assessed the effect of these lipid metabolic changes on various *C. albicans* phenotypes that are frequently studied under laboratory setups. We were able to show that some phenotypic changes were more prominent in YPD compared to YNB, and that the lipid changes due to different media conditions do mask certain phenotypes of *C. albicans*. However, several other tested phenotypes showed no difference between YPD and YNB grown cells. Here we conclude that apart from having an adequate experimental control, it is important to consider that many of the observed changes could be an effect of the media. Therefore, extreme caution should be applied while interpreting lipid-related results. These results demand additional studies under different culture conditions to better understand the lipid metabolism and its corresponding phenotypes. Additionally, certain lipids may be directly contributed by the medium itself and these may affect the *de novo* lipid pool of the cells as well. Of note, the choice of media for growing *C. albicans* or other fungi remains up to the user, but more studies like this could emphasize the consistent use of a particular culture medium by different research groups so that the datasets of independent studies remain comparable.

## Conclusions

In the present study we were clearly able to identify the different lipid profiles of *C. albicans*. Thorough phenotypic analyses showed that the slow growth rate of *C. albicans* in YNB, compared to YPD, correlates with the majority of the observed phenotypic differences between the two media. This study shows an example where the phenotypic difference can be masked by the lipid changes between YPD and YNB. Overall, this study emphasizes the need for a careful examination and interpretation of lipid datasets, as some of the observed changes may be attributed to the media composition.

## Supporting Information

Supplementary Sheet S1 **Molecular species of PGL, SL and SE of YPD and YNB grown** ***C. albicans*** **.** The data is represented as the percentage of total PGL+SL+SE mass spectral signals after normalization to internal standards. The zero values in the table indicate non-significant or undetected values. Error bars indicate ± SEM. (n = 2, for 2 independent analyses of lipid extracts from 2 independent cultures).
(XLS)
